# Leaf dry mass per unit area and leaf pigments underlying the higher stomatal conductance of deciduous species relative to evergreen species in *Dendrobium*

**DOI:** 10.1186/s40529-025-00457-z

**Published:** 2025-03-24

**Authors:** Feng-Ping Zhang, Xiao-Di Zhao, Li-Jun Han, Han-Run Li

**Affiliations:** 1 College of Traditional Chinese Medicine, Yunnan Key Laboratory of Dai and Yi Medicines, Yunnan University of Chinese Medicine, Kunming, Yunnan 650500 China; 2 Kunming Botanical Garden, Kunming Institute of Botany, Chinese Academy of Sciences, Kunming, Yunnan 650201 China

**Keywords:** Leaf pigment, Plant functional traits, Life form, Photosynthesis, Stomatal conductance, *Dendrobium*

## Abstract

**Background:**

Leaf stomatal conductance is an important indicator of photosynthetic capacity. However, stomatal conductance is poorly quantified and rarely explored in the context of the leaf functional traits for epiphytes, particularly when it comes to herbaceous species with different leaf habits (deciduous vs. deciduous species). Here, we investigated leaf stomatal conductance, leaf dry mass per unit area, leaf thickness, stomatal density, abaxial epidermal cell size and pigment contents in 23 *Dendrobium* evergreen and deciduous species from a greenhouse. Our main objectives were to compare differences in all measured traits between evergreen and deciduous species, and to determine the relationships of leaf stomatal conductance with leaf functional traits and leaf pigments.

**Results:**

The results showed that the evergreen species of *Dendrobium* had thicker leaves and higher leaf dry mass per unit area, whereas deciduous species had higher leaf stomatal conductance and higher leaf chlorophyll contents. Leaf stomatal conductance had a negative correlation with leaf thickness, and dry mass per unit area, but a positive correlation with leaf pigment contents. There was a negative correlation between pigment contents and leaf dry mass per unit area.

**Conclusion:**

The results reveal the clear differences in leaf stomatal conductance, leaf functional traits and leaf pigments between deciduous and evergreen *Dendrobium* species, with the form groups showing trait values indicative of less investments in structural components and of more investments in photosynthetic carbon gain. Furthermore, leaf dry mass per unit area and leaf pigments play an important role in shaping leaf stomatal conductance.

## Background

Deciduous plants annually finished a full growth cycle in a comparatively shorter amount of time than evergreen species. Evergreen plants need preserve leaf toughness throughout the year, whereas deciduous species lose their leaves to prevent transpiration. It has been suggested that the two phenological groups differ in the resource trade-off strategies, with deciduous species having a higher photosynthetic capacity demand and evergreen species having a lower photosynthetic capacity (Chabot and Hicks 1982; Kikuzawa 1991; Eamus 1999; Takashima et al. 2004; Flexas et al. 2008; Qi et al. 2021). A global leaf economics spectrum (LES) has been proposed by many researchers about the relationships between leaf photosynthetic capacity, leaf mass per unit area (LMA) and leaf lifespan across species (Reich et al. 1997, 2009; Hikosaka 2004; Wright et al. 2004, 2005; Hallik et al. 2009; Lloyd et al. 2013; Osnas et al. 2013). Leaf pigment content, which is essential to photosynthetic capacity and is a key component of plant photosynthesis and carbon gain, should be tightly associated with leaf functional features within leaf economics spectrum (Li et al. 2013). Few studies, nevertheless, have systematically addressed the relationship between leaf pigments and photosynthetic capacity in relation to plant life history strategies (Matsumoto et al. 2005).

Photosynthetic capacity is one of the key determinants of the amount of carbon acquisition for plants, which is generally larger in deciduous species than evergreen ones (Takashima et al. 2004; Flexas et al. 2008; Qi et al. 2021). Leaf stomatal conductance (g_s_) is an essential component of the cycling and balancing of CO2, water, and energy between plants and the atmosphere. Therefore, stomatal conductance is an indicator of photosynthetic capacity (Wang and Kinoshita 2017). The amount of chlorophyll in leaves is frequently employed as a gauge of their physiological function (Yang et al. 2013). Leaf pigment content was effective for estimating the seasonal variation in stomatal conductance since a drop in chlorophyll concentration causes a decrease in stomatal conductance (Matsumoto et al. 2005). Hesketh (1963) demonstrated, however, that the change in the rate of photosynthesis was unrelated to the amount of chlorophyll. As a result, there is currently disagreement over the association between photosynthetic rate and leaf chlorophyll content.

Leaf functional traits are important because they represent physiological functions. Leaf mass per unit area is one of the key leaf structural traits, and is associated with leaf performance, including leaf lifespan, leaf mechanical strength, photosynthetic rate per leaf mass (Lambers and Poorter 1992; Reich et al. 1997; Westoby 1998; Westoby et al. 2002; Wright et al. 2004; Sancho-Knapik et al. 2021). It has been suggested that there was a closely negatively relationship between photosynthetic capacity and leaf mass per unit area (Pearce et al. 1969; Reich et al. 1997; Wright et al. 2004; Shipley et al. 2006). Compared to deciduous species, evergreens typically have lower net photosynthesis, despite having larger leaf mass per unit area and longer leaf lifespans (Zhao et al. 2017). Additionally, shade leaves had lower leaf dry mass per unit area (LMA), an important leaf structural parameter (Reich et al. 1997; Wright et al. 2004) and higher amounts of the mass-based photosynthetic pigments (e.g. Chlorophyll) than sun leaves, suggested that shade leaves under low light invested less in structural components and more in light harvesting (Matsubara et al. 2009). Regardless of environmental variations of life form, there is leaf mass per unit area and leaf pigment concentrations (Li et al. 2013). The majorities of previous studies on distinctions and relationships in leaf functional traits between evergreen and deciduous species have focused on wood species (Fu et al. 2012; Li et al. 2013; Guan et al. 2023; Chen et al. 2023a, b; Huang and Zeng 2023), but few studies have been conducted on herbaceous species that are linked to leaf habits, such as evergreen and deciduous (Li et al. 2022).

The genus *Dendrobium* (Orchidaceae, subtribe Dendrobiinae) is a perennial herb, is one of the largest groups of epiphytic orchids in the world, with roughly 1200–1500 species mainly found in tropical Asia and Oecania (Cribb and Govaerts 2005; Wood 2006; Zhu et al. 2009; Pridgeon et al. 2014; Xiang et al. 2016). *Dendrobium* species are among the most popular orchids (referred to as “flowers of Father’s Day”) in the world’s horticulture commerce because of their beautiful flowers (Teixeirada Silva et al. 2016). Furthermore, the pseudobulbs of certain *Dendrobium* species-known as “Fengdou” in Chinese, which had significant commercial importance in China as key components of traditional herbal medications used in oriental medicine for hundreds of years (Bao et al. 2001). Moreover, according to the IUCN Red list of higher plants in China (http://www.zhb.gov.cn/gkml/hbb/bgg/201309/t20130912_260061.htm), many wild *Dendrobium* species are in extreme danger of going extinct because of their slow growth and germination rates, habitat destruction, and overexploitation (Niu et al. 2017). The majority of *Dendrobium* species are usually epiphytic and grow on the trees of evergreen broad-leaved forests keystone groups (Tsi 1999; Wood 2006; Zhu et al. 2009; Xiang et al. 2016). However, few studies have been done on the parameters of leaf pigment contents, leaf mass per unit area and stomatal conductance in *Dendrobium*, despite the fact that most recent studies have been focused on their molecular systematics (Xiang et al. 2013, 2016; Zhu et al. 2018; Li et al. 2020), functional genes (Deng et al. 2023), chemical component and functions (He et al. 2022, 2024) and so forth.

In the present study, we examined leaf chlorophyll contents, leaf mass per unit area, leaf stomatal conductance, leaf thickness, stomatal density, and abaxial epidermal cell size of 16 deciduous and seven evergreen *Dendrobium* species from the greenhouse. Our objectives were to determine: (1) whether the 23 species of *Dendrobium* from the two leaf habit groups differ in terms of leaf pigment contents, leaf thickness, leaf mass per unit area, leaf stomatal conductance, and stomatal density, and abaxial epidermal cell size. We hypothesize that different leaf habits reflect alternative resource trade-off strategies. In particular, the evergreen *Dendrobium* species have thicker leaves and higher leaf mass per unit area, while deciduous *Dendrobium* species have higher leaf stomatal conductance and higher leaf pigment contents. (2) whether leaf stomatal conductance is related to leaf pigments content, leaf thickness and leaf mass per unit area, and stomatal density, and abaxial epidermal cell size as well as whether these leaf functional traits correlated.

## Materials and methods

### Plant materials

Our study was carried out at the Kunming Botanical Garden, Kunming Institute of Botany (KIB), Chinese Academy of Sciences (25°10′N, 102°41′E, 1900 m a.s.l.), located at Kunming City, Yunnan Province, in southwestern China. All plant materials of the 23 *Dendrobium* species (Table 1) were cultivated in a greenhouse with relative humidity of 60–70% and temperatures of 20–25℃ during the day and 10–15℃ at night for three years at Kunming Botanical Garden, and they were watered as needed.

**Table 1:** The leaf phenological trait of the studied 23 *Dendrobium* species

Species	Leaf phenological types	Altitude (m)
*D*. *cariniferum*	Evergreen	1100–1700
*D*. *trigonopus*	Evergreen	1150–1600
*D*. *chrysotoxum*	Evergreen	520–1620
*D*. *densiflorum*	Evergreen	420–1000
*D*. *harveyanum*	Evergreen	1100–1700
*D*. *jenkinsii*	Evergreen	700–1300
*D*. *terminale*	Evergreen	850–1080
*D*. *stuposum*	Deciduous	1800
*D*. *devonianum*	Deciduous	1850
*D*. *falconeri*	Deciduous	800–1900
*D*. *wattii*	Deciduous	2000
*D*. *ellipsophyllum*	Deciduous	1100
*D*. *aduncum*	Deciduous	700–1000
*D*. *crepidatum*	Deciduous	1000–1800
*D*. *crystallinum*	Deciduous	540–1700
*D*. *flexicaule*	Deciduous	1200–2000
*D*. *wardianum*	Deciduous	1350–1900
*D*. *chrysanthum*	Deciduous	700–2500
*D*. *moniliforme*	Deciduous	1000–1300
*D*. *hancockii*	Deciduous	700–1500
*D*. *linawianum*	Deciduous	400–1500
*D*. *brymerianum*	Deciduous	1100–1900
*D*. *pendulum*	Deciduous	1050–1600

### Leaf thickness, leaf dry mass per unit area, and leaf anatomical traits

Leaf thickness of mature leaves from six individuals were measured by an outside micrometer. Leaf areas (cm^2^) were also measured from other six mature leaves with with a Li-Cor 3000 A area meter (Li-Cor, Inc., USA). Subsequently, these leaves were oven-dried at 70℃ for 48 h to obtain their dry weights (DW). The leaf dry mass per unit area (LMA, g m^− 2^) was calculated as the ratio of leaf dry mass to leaf area. For calculating stomatal density, the abaxial epidermis from the middle part of six mature leaves were coated with a thin layer of colorless, transparent nail polish, the films were transferred to microscope slides after drying. The stomata on these films were captured on a under a light microscope (Leica DM2500, Germany). Thirty randomly selected digital images for each species were used to observe epidermal cell and count the stomatal number (Yang et al. 2016, 2018), and measure using the ImageJ software (National Institutes of Health, Bethesda, MD, United States). Epidermal cell size was subsequently calculated as in Carins Murphy et al. (2016) using the following equation: Epidermal cell size = (1-(mean stomatal size х stomatal density)/epidermal cell density. Stomatal density (SD) was calculated as the number of stomata per leaf area.

### Leaf stomatal conductance and chlorophyll contents

The leaf stomatal conductance (g_s_) was measured between 09:00 to 11:30 from six selected individuals per species, using a Li-600 portable porometer (Li-Cor, Inc., USA). Leaf chlorophyll (Chl) was extracted in NN-dimethylformamide and measured with a spectrophotometer (UV-2550, Shimadzu, Kyoto, Japan) following the method of Inskeep and Bloom (Inskeep and Bloom 1985). Six fully developed and healthy leaves from six individuals for each species were placed in a cold box with ice and then taken to laboratory. 0.15 g of leaves were weighed and cut into fragments of 0.5 cm × 0.5 cm. They were then placed in a centrifuge tube (15 ml) and diluted to 10 mL with N, N-dimethylformamide. The tube was left in the dark for 48 h, and absorbance values were measured using a UV spectrophotometer at 450 nm, 647 nm, and 664.5 nm. The content of chlorophyll a (Chla), chlorophyll b (Chlb), total chlorophyll (Chl (a + b), and carotenoids (Car) were calculated using the following formula (µg ml^− 1^):

Chl a = 12.70A_664.5_-2.79A_647_.

Chl b = 20.70A_647_-4.62A_664.5_.

Chl a + b = 17.9A_647_ + 8.08A_664.5_.

Car = 4.695A_450_-0.268 (Chl a + Chl b).

Subsequently, the chlorophyll content per mass (mg g^− 1^) was calculated based on the leaf mass and solution volume.

### Data analysis

To determine differences between evergreen and deciduous *Dendrobium* species in terms of leaf stomatal conductance, chlorophyll content, leaf thickness, leaf dry mass per unit area, and stomatal density, and abaxial epidermal cell size, one-way ANOVA tests after testing for the normality and homogeneity of variables were conducted. The associations of leaf stomatal conductance with leaf pigment and leaf functional traits were tested by regression analyses. Correlations between all traits were conducted using Pearson’s correlation tests across all species. To further explore the relative importance of leaf functional traits and leaf pigments to the variation of leaf stomatal conductance, we applied a hierarchical partition method to split the influence of leaf functional traits and leaf pigments on leaf stomatal conductance using the R package glmm.hp (Lai et al. 2022). A principal component analysis (PCA) based on the nine traits measured was applied to test the coordination among traits in the 23 *Dendrobium* species. The species loading based on the principal components (PCs) were plotted using the package ‘factoextra’ in v.4.3.0 (R Core Team 2020).

## Results

### Comparison of stomatal conductance, chlorophyll content, and leaf functional traits between deciduous and evergreen *Dendrobium* species

The *Dendrobium* species used in the present study exhibited significantly variations in stomatal conductance, chlorophyll, carotenoid contents, as well as leaf functional traits (Table 2, Table S1). Among evergreen *Dendrobium* species, a very large range of a extending from 403.06 μm to 1312.10 μm in the leaf thickness was observed. Evergreen *Dendrobium* species varied 5.00-fold in the stomatal conductance, from 0.004 mol m^− 2^ s^− 1^ to 0.02 mol m^− 2^ s^− 1^. The stomatal density varied 2.70-fold across the studied *Dendrobium* species, from 26.98 mm^− 2^ to72.96 mm^− 2^. Abaxial epidermal cell size varied from 1.28 mm^2^ × 10^− 3^ to 4.06 mm^2^ × 10^− 3^. Leaf dry mass per unit area varied 2.31-fold, from 90.20 g m^− 2^ to 208.43 g m^− 2^. Chlorophyll a varied 1.43-fold, from 0.37 mg g^− 1^ to 0.53 mg g^− 1^. Chlorophyll b varied 2.29-fold, from 0.14 mg g^− 1^ to 0.32 mg g^− 1^. Chlorophyll a + b varied from 0.51 mg g^− 1^ to 0.75 mg g^− 1^. Carotenoid varied 2.50-fold, from 0.04 mg g^− 1^ to 0.10 mg g^− 1^.

**Table 2:** Quantification of the measured traits for 23 the studied *Dendrobium* species

Groups	Traits	Mean ± SE	Min	Max	CV (%)
Evergreen	*g*_s_	0.01 ± 0.002	0.004	0.02	44.34
	SD	53.14 ± 6.89	26.98	72.96	34.28
	AECS	2.34 ± 0.39	1.28	4.06	44.70
	LT	633.28 ± 119.40	403.06	1312.10	49.89
	LMA	137.78 ± 16.24	90.20	208.43	31.18
	Chl a	0.46 ± 0.02	0.37	0.53	12.99
	Chl b	0.21 ± 0.02	0.14	0.32	25.91
	Chl a + b	0.67 ± 0.03	0.51	0.75	12.58
	Car	0.07 ± 0.007	0.04	0.10	26.86
Deciduous	*g*_s_	0.04 ± 0.002	0.02	0.06	27.77
	SD	63.34 ± 4.88	39.14	97.57	30.81
	AECS	1.78 ± 0.15	1.04	2.82	34.58
	LT	303.63 ± 25.61	174.53	466.66	33.74
	LMA	57.88 ± 4.96	29.01	101.08	34.35
	Chl a	1.29 ± 0.11	0.78	2.43	35.07
	Chl b	0.50 ± 0.04	0.32	0.84	34.57
	Chl a + b	1.80 ± 0.15	1.12	3.27	34.55
	Car	0.15 ± 0.01	0.08	0.25	38.79

g_s_, stomatal conductance (mol m^− 2^ s^− 1^); SD, stomatal density (mm^− 2^); AECS, abaxial epidermal cell size (mm^2^ × 10^− 3^); LT, leaf thickness (µm); LMA, leaf dry mass per unit area (g m^− 2^); Chl a (mg g^− 1^), Chlorophyll a (mg g^− 1^); Chl b, Chlorophyll b (mg g^− 1^); Chl a + b, Chlorophyll a + b (mg g^− 1^); Car, Carotenoid (mg g^− 1^)

Deciduous species had the lowest leaf dry mass per unit area (29.01 g m^− 2^) and the smallest leaf thickness (174.53 μm) and the largest leaf stomatal conductance (0.06 mol m^− 2^ s^− 1^) (Table 2). Leaf stomatal density ranged from 39.14 to 97.57 mm^− 2^ among the deciduous species, abaxial epidermal cell size varied from 1.04 to 2.82 mm^2^ × 10^− 3^, chlorophyll a varied 3.12-fold from 0.78 mg g^− 1^ to 2.43 mg g^− 1^, chlorophyll b varied 2.63-fold from 0.32 mg g^− 1^ to 0.84 mg g^− 1^, chlorophyll a + b varied 2.92-fold from 1.12 mg g^− 1^ to 3.27 mg g^− 1^. Carotenoid varied 3.13-fold, from 0.08 mg g^− 1^ to 0.25 mg g^− 1^ among the deciduous species.

**Fig. 1:**
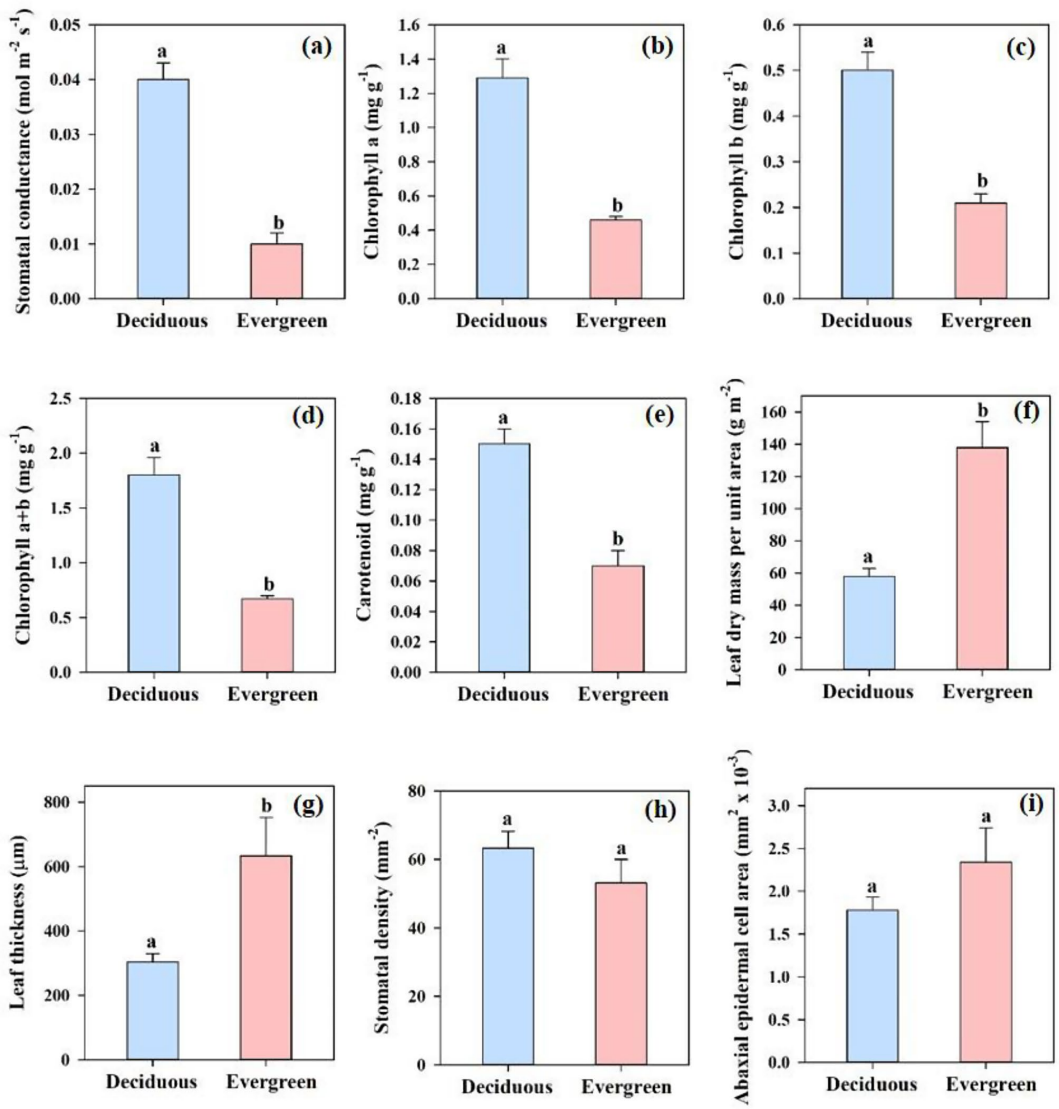
Difference in leaf stomatal conductance (**a**), chlorophyll a (**b**), chlorophyll b (**c**), chlorophyll a + b (**d**), carotenoid contents (**e**), leaf dry mass per unit area (**f**), leaf thickness (**d**), stomatal density (**h**), and abaxial epidermal cell size (**i**). One-way ANOVA was used to test the differences in measured traits between evergreen and deciduous *Dendrobium* species. The different lowercase letters above the columns indicates statistically significant differences (*P* < 0. 05) between deciduous and evergreen species. The same lowercase letters above the columns indicates non-significant differences (*P* > 0. 05) between deciduous and evergreen species

Evergreen and deciduous *Dendrobium* species also differed significantly in the stomatal conductance, leaf thickness, leaf dry mass per unit area, chlorophyll a, chlorophyll b, chlorophyll a + b and carotenoid (Fig. 1). Compared with the evergreen *Dendrobium* species, the deciduous *Dendroibium* species had significantly higher leaf stomatal conductance and leaf pigment contents. The evergreen species had significantly higher leaf dry mass per unit area and thicker leaf than those of the deciduous species. However, there was no significant difference in either stomatal density (*P* = 0.25) or abaxial epidermal cell size (*P* = 0.12) between evergreen and deciduous *Dendrobium* species (Fig. 1).

### Relationships between stomatal conductance, chlorophyll content, and leaf functional traits

**Fig. 2:**
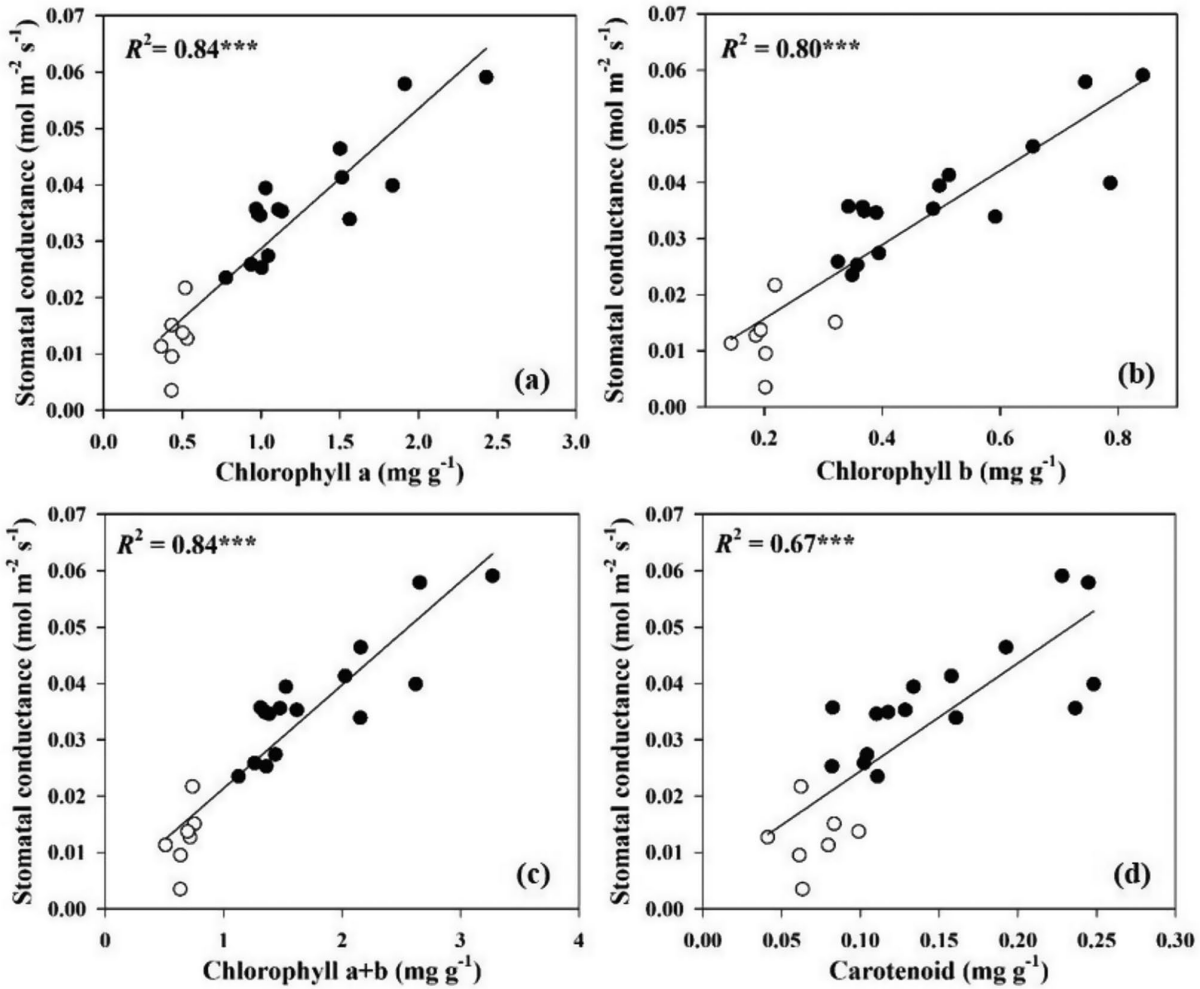
Relationships of stomatal conductance with chlorophyll a (**a**), chlorophyll b (**b**), chlorophyll a + b (**c**) and carotenoid contents (**d**) for the 23 studied *Dendrobium* species. The filled and open circles represent deciduous and evergreen *Dendrobium* species, respectively. Coefficient of determination (*R*²) and regression lines are shown in solid lines. Statistical significances are shown.*, *P* < 0.05; **, *P* < 0.01; ***, *P* < 0.001

**Fig. 3:**
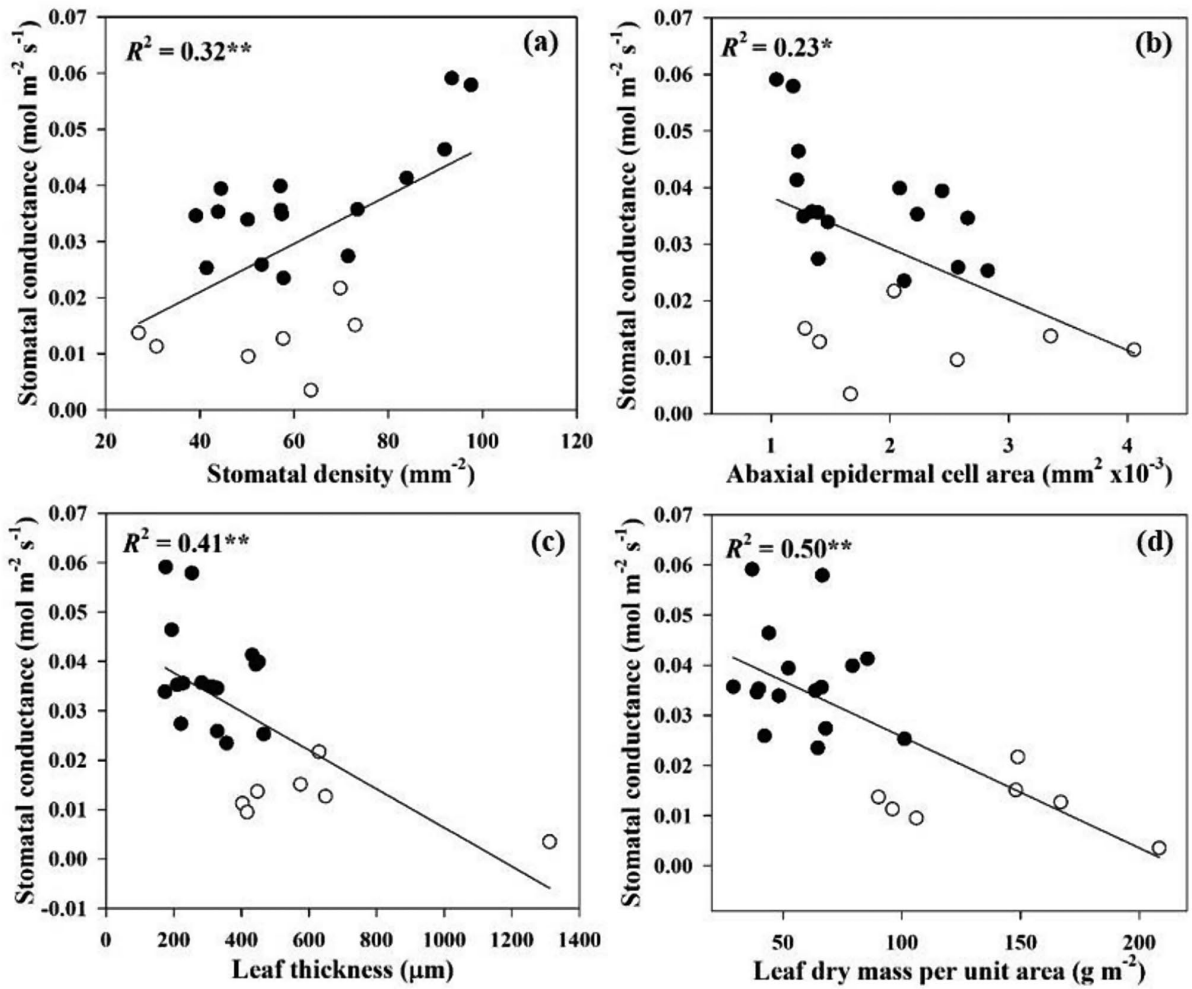
Relationships of stomatal conductance with stomatal density (**a**), Abaxial epidermal cell size (**b**), leaf thickness (**c**) and leaf dry mass per unit area (**d**) for the 23 studied *Dendrobium* species. The filled and open circles represent deciduous and evergreen *Dendrobium* species, respectively. Coefficient of determination (*R*²) and regression lines are shown in solid lines. Statistical significances are shown.*, *P* < 0.05; **, *P* < 0.01; ***, *P* < 0.001

**Fig. 4:**
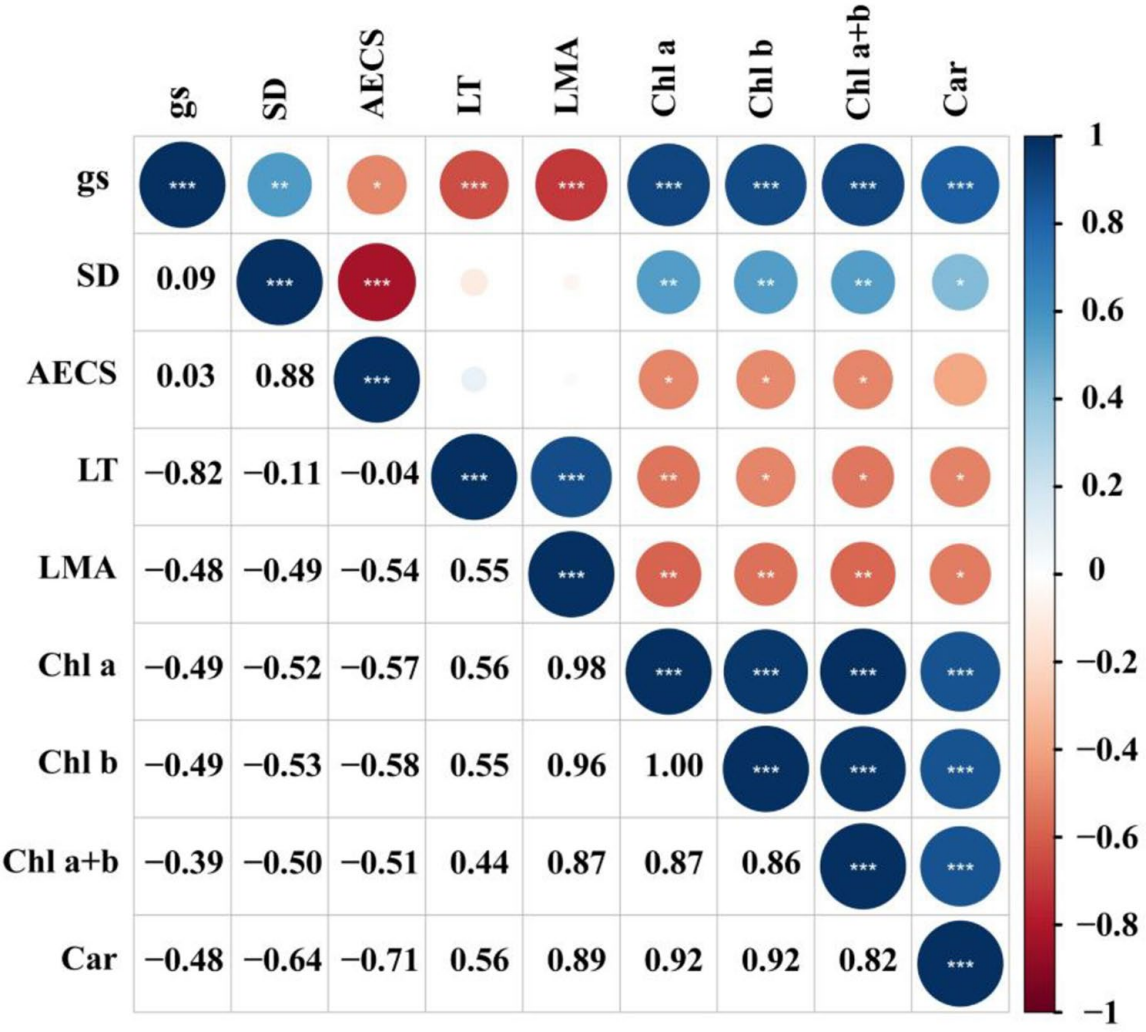
Pearson’s correlation coefficient analysis among stomatal conductance, leaf functional traits, chlorophyll and carotenoid content for the 23 studied *Dendrobium* species. Circle sizes and colors represent the significance and correlation coefficient (***r***). Significant levels are shown. *P < 0.05; **P < 0.01; ***P < 0.001. g_s_, stomatal conductance); SD, stomatal density; AECS, abaxial epidermal cell size; LT, leaf thickness; LMA, leaf dry mass per unit area; Chl a, Chlorophyll a; Chl b, Chlorophyll b; Chl a + b, Chlorophyll a + b; Car, Carotenoid

**Fig. 5:**
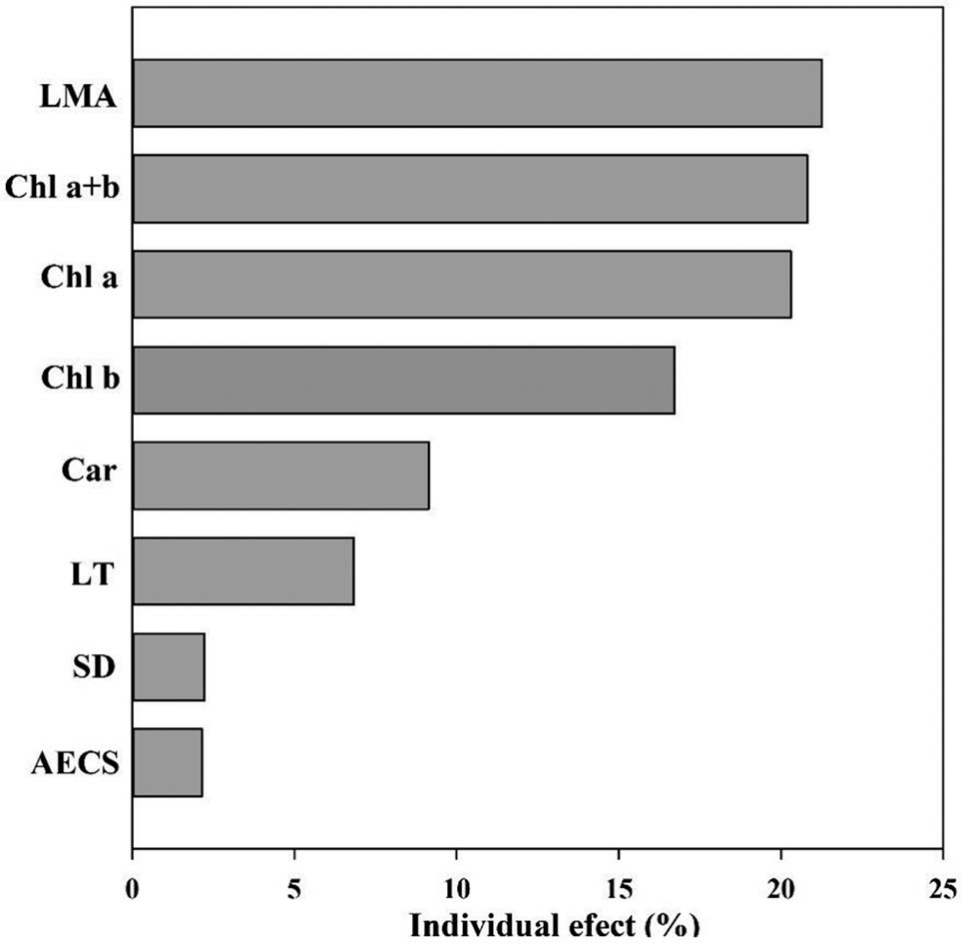
The relative importance of leaf functional traits and leaf pigments in explaining variances in leaf stomatal conductance. Each bar shows variance explained by theindependent effects of each variable. Detailed results are given in Tables S2

In order to determine whether the stomatal conductance can be predicted from chlorophyll, carotenoid contents, the relationships between stomatal conductance and chlorophyll and carotenoid contents were investigated. Across all *Dendrobium* species, the stomatal conductance was linearly correlated with chlorophyll a (*R*^2^ = 0.84, *P* < 0.001), chlorophyll b (*R*^2^ = 0.80, *P* < 0.001), chlorophyll a + b (*R*^2^ = 0.84, *P* < 0.001), and carotenoid (*R*^2^ = 0.67, *P* < 0.001) (Fig. 2). We also investigated leaf functional traits, there was a tight positive correlation between the stomatal conductance and stomatal density (*R*^2^ = 0.32, *P* < 0.01) (Fig. 3).However, the negative correlations of the stomatal conductance with abaxial epidermal cell size (*R*^2^ = 0.23, *P* < 0.05), leaf thickness (*R*^2^ = 0.41, *P* < 0.01), and leaf dry mass per unit area were observed (*R*^2^ = 0.50, *P* < 0.01). There were significantly positive correlation among chlorophyll a, chlorophyll b, chlorophyll a + b, and carotenoid across all the studied species (Fig. 4). Leaf dry mass per unit area, chlorophyll, carotenoid contents, leaf thickness, stomatal density, and abaxial epidermal cell size showed significant association with stomatal conductance when each predictor was considered alone, leaf dry mass per unit area had a larger contribution to stomatal conductance (Fig. 5).

**Fig. 6:**
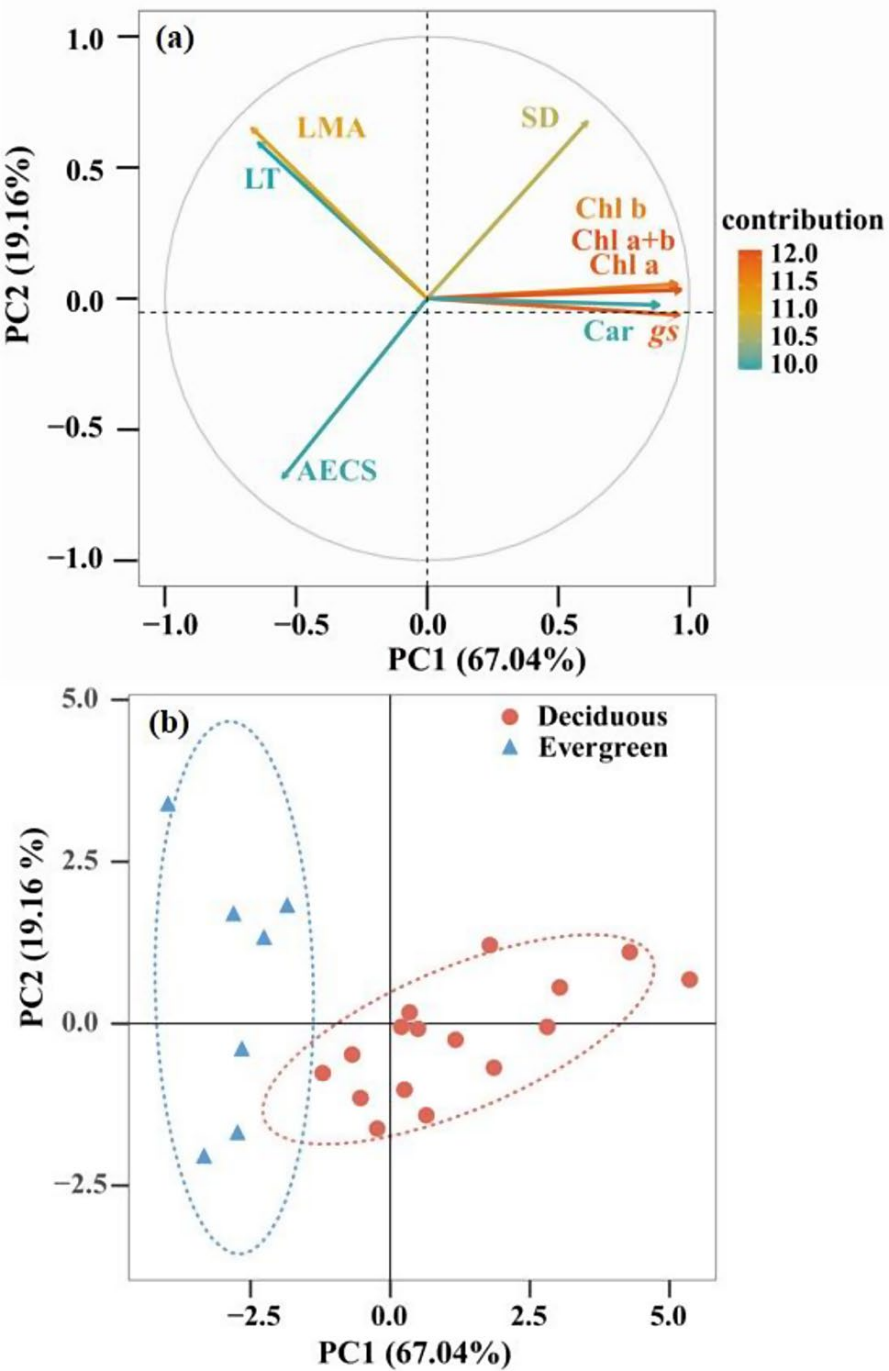
PCA of the 23 *Dendrobium* species studied based on 9 leaf traits. (**a**)The loading of the first two PCs, and (**b**) scores of each species along PC1 and PC2 for evergreen species (red circles) and deciduous *Dendrobium* species (blue triangles). g_s_, stomatal conductance); SD, stomatal density; AECS, abaxial epidermal cell size; LT, leaf thickness; LMA, leaf dry mass per unit area; Chl a, Chlorophyll a; Chl b, Chlorophyll b; Chl a + b, Chlorophyll a + b; Car, Carotenoid

**Fig. 7:**
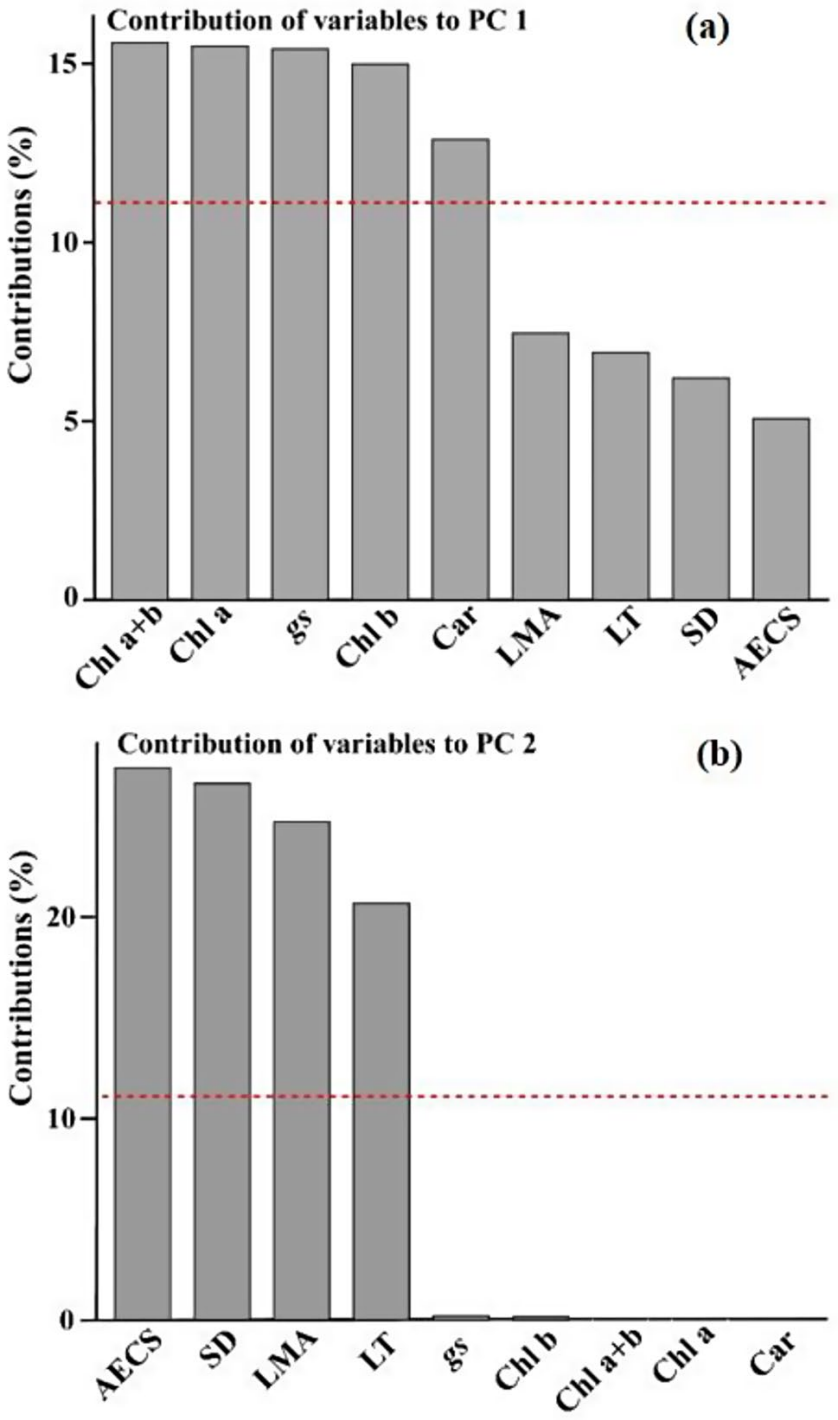
The contribution of variables to the first and second PC based on the PCA analysis, respectively. The red dashed lines in (**a**) and (**b**) indicate the average contribution of each variable to the corresponding PC. g_s_, stomatal conductance); SD, stomatal density; AECS, abaxial epidermal cell size; LT, leaf thickness; LMA, leaf dry mass per unit area; Chl a, Chlorophyll a; Chl b, Chlorophyll b; Chl a + b, Chlorophyll a + b; Car, Carotenoid

The principal component analysis (PCA) base on nine traits for the 23 *Dendrobium* species indicated that the first and second component axes explained 67.04% and 19.16% of the total variations, respectively (Fig. 6). The first PC was mainly explained by the chlorophyll a + b content (15.59%), chlorophyll a content (15.49%), stomatal conductance (15.41%), chlorophyll b content (14.99%), and carotenoid content (12.88%) (Fig. 7). The second PC was contributed mainly by the abaxial epidermal cell size (27.41%), stomatal density (26.63%), leaf dry mass per unit area (24.73%), and leaf thickness (20.69%). Furthermore, evergreen and deciduous *Dendrobium* species were separated by the PC1 (*P* = 0.03, *t* = -5.51) axes (Fig. 6).

## Discussion

In our study, we examined nine leaf traits for 23 *Dendrobium* species grown in the greenhouse. The outcome broadens our understanding of photosynthetic capacity, chlorophyll content and leaf functional traits in *Dendrobium* species. We determined whether variations in leaf pigments and important functional traits could mechanistically explain why deciduous *Dendrobium* species are frequently more likely than evergreen species to attain higher photosynthetic capacity. We also determined whether maintenance costs that are dependent on chlorophyll contents and leaf dry mass per unit area are likely to be a significant factor in the variation of leaf stomatal conductance across the studied *Dendrobium* species.

### Differences in stomatal conductance, pigment content, and leaf functional traits between deciduous and evergreen *Dendrobium* species

Our findings demonstrated that the evergreen and deciduous *Dendrobium* species differed significantly in leaf stomatal conductance, chlorophyll content, and leaf functional traits. The evergreen species had higher leaf dry mass per unit area and thicker leaves, while deciduous species had significantly higher stomatal conductance and higher leaf chlorophyll contents. These results support our first hypothesis that different leaf habits reflect different resource trade-off strategies. Our finding aligns with findings of earlier investigations (Reich et al. 1997; Castro-Díez et al. 2000; Westoby et al. 2002; Wright and Westoby 2002; Wright et al. 2004; Takashima et al. 2004; Li et al. 2013; Huang et al. 2021; Sun et al. 2023). The higher photosynthetic capacity might have been used by deciduous species to make up for their comparatively short leaf lifespans (Matsumoto et al. 2005). Evergreen species should invest less photosynthate per increase in leaf dry mass per unit area if their leaf pigments are better protected than those of deciduous species (Poorter et al. 2009; Li et al. 2015). Evergreen species maintain functional leaves throughout the year to compensate for their reduced photosynthetic capacity (Fu et al. 2012).

The leaf economic spectrum represents a set of trade-offs of leaves, such as those between leaf lifespans and leaf dry mass per unit area, are represented by the leaf economic spectrum. A high leaf turnover rate combined with low construction costs is indicated by a low leaf dry mass per unit area (Wright et al. 2004; Freschet et al. 2010; Dong et al. 2020; Li et al. 2024). Notably, evergreen species have substantially greater leaf thickness and leaf dry mass per unit area than deciduous *Dendrobium* species, which may imply that evergreen species with longer leaf life appear to devote more resources to their leaf cell walls to improve tissue toughness (Chen et al. 2020). Our result is in agreement with other previous findings (Fu et al. 2012; Li et al. 2013; Sancho-Knapik et al. 2021; Guan et al. 2023). For instance, compared to deciduous oak species, evergreen oak species have significantly thicker leaves with a higher leaf dry mass per unit area (Sancho-Knapik et al. 2021). It is commonly believed that deciduous species’ lower leaf dry mass per unit area and evergreens’ higher leaf dry mass per unit area are adaptation and growth strategies. Evergreen plants, for example, have longer leaf lifespans due to their larger leaf dry mass per unit area (Garkoti and Singh 1994). Conversely, deciduous species may benefit from cheaper leaf construction costs due to their lower leaf construction (Givnish 1988; Walters and Reich 1999).

### Correlations between stomatal conductance, chlorophyll contents, and leaf functional traits

Over the past few decades, numerous researchers have examined the relatinships between various leaf functional traits, including respiration, phosphorus and nitrogen content, photosynthetic capacity, leaf lifespan, leaf dry mass per unit area and leaf lifespan, as a model for large-scale research (Reich et al. 1997; Wright et al. 2004). The trade-offs between leaf economics spectrum and pigment contents are shown in the present study. Traits related to leaf economics spectrum (leaf dry mass per unit area and leaf thickness) clustered together on the negative side of PC axis 1, while traits associated with photosynthetic capacity (stomatal conductance) and pigments (chlorophyll a, chlorophyll b, chlorophyll a + b and carotenoid) clustered together on the positive site. The correlation between leaf pigment, leaf dry mass pe area and leaf gas exchange in the present study was demonstrated in the present study, which was in line with previous studies (Li et al. 2013; Chen et al. 2023a, b). Specifically, our study reveals that leaf stomatal conductance was positively correlated with chlorophyll a, chlorophyll b, chlorophyll a + b and carotenoid contents across the studied *Dendrobum* species. Species with higher leaf dry mass per unit area and thicker leaves usually had lower photosynthetic capacity (Reich et al. 1997; Wright et al. 2004, 2005), which suggest that the stomatal conductance variability depended significantly on the concentration of chlorophyll (Matsumoto et al. 2005).

We were surprised to found that leaf stomatal conductance was negatively correlated with leaf dry mass per unit area and leaf thickness. Because the rate of leaf photosynthesis is positively correlated with chlorophyll (Emerson 1929), leaf chlorophyll concentration is expected to be negatively correlated with LMA. The negative relationship between leaf chlorophyll content and LMA has been documented in several studies (Woodward 1979; Ruhland and Day 1996; Hallik et al. 2009), and it is frequently proposed that higher leaf dry mass per unit area shields chlorophyll from environmental stresses. The length of the CO2 diffusion path may be impacted by the leaf thickness and the thickness of cell wall and leaves, which could limit potentially CO2 diffusion to chloroplasts and lower photosynthetic rates (Flexas et al. 2012; Tosens et al. 2012; Onoda et al. 2017; Yang et al. 2018). Thus, the correlation between stomatal conductance and the key leaf functional traits supports leaf economics theory that photosynthetic capacity is associated with the leaf economics spectrum (Wright et al. 2004; Li et al. 2024). These relationships showed that the leaf economic traits were linked to the internal diffusion of CO2 conductance and photosynthetic performances of species within *Dendrobium*. We demonstrated a negative correlation between leaf dry mass per unit area and leaf pigment contents. Our finding is in agreement with the results of Matsubara et al. (2009), who documented a trade-off between leaf dry mass per unit area and leaf pigment concentrations. Therefore, we proposed that leaf dry mass per unit area and leaf pigment should be taken into consideration when attempting to predict photosynthetic capacity, more plant species should be included in future experimental observations.

## Conclusion

In summary, evergreen and deciduous *Dendrobium* species differed in stomatal conductance, leaf thickness, leaf dry mass per unit area as well as leaf pigment contents. Deciduous species exhibited higher leaf pigment contents, thinner leaf and lower leaf dry mass per unit area in order to sustain a higher photosynthetic capacity. Conversely, evergreen species had lower leaf pigment contents, thicker leaf and higher leaf dry mass per unit area and lower photosynthetic capacity. Leaf stomatal conductance was significantly correlated with leaf pigment contents, leaf dry mass per unit area, leaf thickness, and stomatal density, and abaxial epidermal cell size. In particular, leaf dry mass per unit area and leaf pigment contents which are important and easily measurable traits can be used as indicators of leaf stomatal conductance. Our study highlighted the important role of leaf dry mass per unit area and leaf pigment contents in explaining the differences of photosynthetic capacity between deciduous species and evergreen *Dendrobium* species.

## Data Availability

All data has been provided with the manuscript.
